# Urban Livestock Keeping in the City of Nairobi: Diversity of Production Systems, Supply Chains, and Their Disease Management and Risks

**DOI:** 10.3389/fvets.2017.00171

**Published:** 2017-10-25

**Authors:** Pablo Alarcon, Eric M. Fèvre, Patrick Muinde, Maurice K. Murungi, Stella Kiambi, James Akoko, Jonathan Rushton

**Affiliations:** ^1^Royal Veterinary College, University of London, London, United Kingdom; ^2^Leverhulme Centre for Integrated Research in Agriculture and Health, London, United Kingdom; ^3^Institute for Infection and Global Health, University of Liverpool, Liverpool, United Kingdom; ^4^International Livestock Research Institute (ILRI), Nairobi, Kenya; ^5^University of Nairobi, Nairobi, Kenya

**Keywords:** urban livestock, supply chain, disease management, food safety, Nairobi, gender, risk practices

## Abstract

Urban livestock keeping in developing cities have an important role in food security and livelihoods but can also pose a significant threat to the environment and health of urban dwellers. The aim of this study was to identify the different livestock systems in Nairobi, their supply chains, and their management and food safety risks. Seven focus group discussions with livestock production officers in charge of each major Nairobi sub-county were conducted. Data were collected on the type of systems existing for each livestock species and their supply chains, disease management, food safety risks, and general husbandry and gender factors. Supply chain flow diagrams and thematic analysis of the data was done. Results of the study show a large variability of livestock keeping in Nairobi. The majority were small scale with: <5 dairy cows, 1–6 dairy goats, <10 small ruminants, <20 pigs, 200–500 broilers, 300–500 layers, <10 indigenous chickens, or <20 rabbits. Beef keeping was mainly described as a “by the way” system or done by traders to fatten animals for 3 month. Supply chain analysis indicated that most dairy farmers sold milk directly to consumers due to “lack of trust” of these in traders. Broiler and pig farmers sold mainly to traders but are dependent on few large dominating companies for their replacement or distribution of products. Selling directly to retailers or consumers (including own consumption), with backyard slaughtering, were important chains for small-scale pig, sheep and goat, and indigenous chicken keepers. Important disease risk practices identified were associated with consumption of dead and sick animals, with underground network of brokers operating for ruminant products. Qualified trained health managers were used mainly by dairy farmers, and large commercial poultry and pig farmers, while use of unqualified health managers or no treatment were common in small-scale farming. Control of urban livestock keepers was reported difficult due to their “feeling of being outlaws,” “lack of trust” in government, “inaccessibility” in informal settlements, “lack of government funding,” or “understaffing.” Findings are useful for designing policies to help to control urban livestock production and minimize its associated health and environment risks.

## Introduction

Urban agriculture is a dynamic concept that comprises a variety of livelihood systems ranging from subsistence production and processing at the household level to more commercialized agriculture. However, many urban farmers around the world operate without formal recognition of their main livelihood activity and lack the structural support of proper municipal policies and legislation (1). The attention given to urban agriculture has grown quickly over the past decades with the formation of an urban agriculture advisory committee by the United Nations Development Program in 1991. Mireri (2) classified urban farmers into three categories: (1) urban inhabitants who rely on farming as an important source of food; (2) commercial urban farmers who are formally employed and engaged in farming to supplement their hitherto low wages; and (3) those doing farming as their employment due to a weak economic base or lack of appropriate skills to participate in the modern sector. In addition, some people in urban areas keep animals for traditional purposes or as hobby. All these urban livestock keepers can play an important role in food security, but can also represent an important risk of pathogens transmission (zoonotic and non-zoonotic) and environmental contamination (1, 3, 4).

Nairobi, with 3.4 million inhabitants, is one of the fastest-growing cities in Africa with increasing demand for land and animal source products (5). The conversion and encroachment of potential agricultural lands into urban and peri-urban residential uses is leading to rapid transformations of the agricultural production (6). Today, regardless of farming being prohibited within city boundaries, there is a significant population of livestock (7). According to the 2013 report produced by the Kenyan Ministry of Livestock and Development (MoLD), the livestock population in the city was around 1.3 million (8). Crude biomass estimations indicate that there was 0.22 kg of livestock biomass per 1 kg of human biomass (0.11 kg of pigs, 0.09 kg of dairy cattle, 0.2 kg of beef, sheep and goats, 0.01 kg of poultry).^1^ Poultry (with over 880,000 birds, half of them broilers) and pigs represented, however, the largest number of livestock in the city. In the period 2009–2012, the population of broilers in Nairobi has doubled, the population of layer birds has increased by 34% and the population of pigs has increased by 56% (8). Urban dairy cattle produced almost 4.5 million kilogram of milk per year, with a 4 and 14% increase in production in 2012 and 2011, respectively. Dairy, broiler, and egg production represented the priority commercial enterprise among livestock keepers in most parts of the city. Rabbit and dairy goat are emerging urban productions, while the sheep population rose 15% in 2012. Despite the overall increase in urban livestock population in Nairobi, there is a lack of comprehensive studies describing the type of livestock systems in the city, the value chains used, their role and their animal health and food safety management. For this, thematic qualitative research methods are useful as they allow exploring and identifying the diversity of systems and factors, and avoid restricting findings to predetermined knowledge. Furthermore, given the large size of the city and the wide range of livestock species raised, focus group discussions (FGDs) with key informants represent the most efficient approach to capture an overview of urban livestock keeping that can then be used for more detailed and focused research studies. For this, the livestock production officers (LPOs) represent a potential group of key informants. These are public administrators within the Ministry of Livestock Development whose jurisdiction is to supervise, give advice, and provide extension services on husbandry and farm practices to livestock keepers. These officers are, therefore, routinely exposed to the different types of livestock keepers in the city, giving them an important field experience and overall understanding of these urban systems, as shown in their 2013 report on livestock production in Nairobi (8).

The present study aims, through focus groups with Nairobi LPOs, to (1) identify and quantify the type of livestock keepers in Nairobi, (2) map their supply chains, (3) describe their main husbandry and gender patterns, and (4) assess their principal animal health management and food safety risk practices.

## Materials and Methods

### The Approach and Selection of Participants

**Figure 1:**
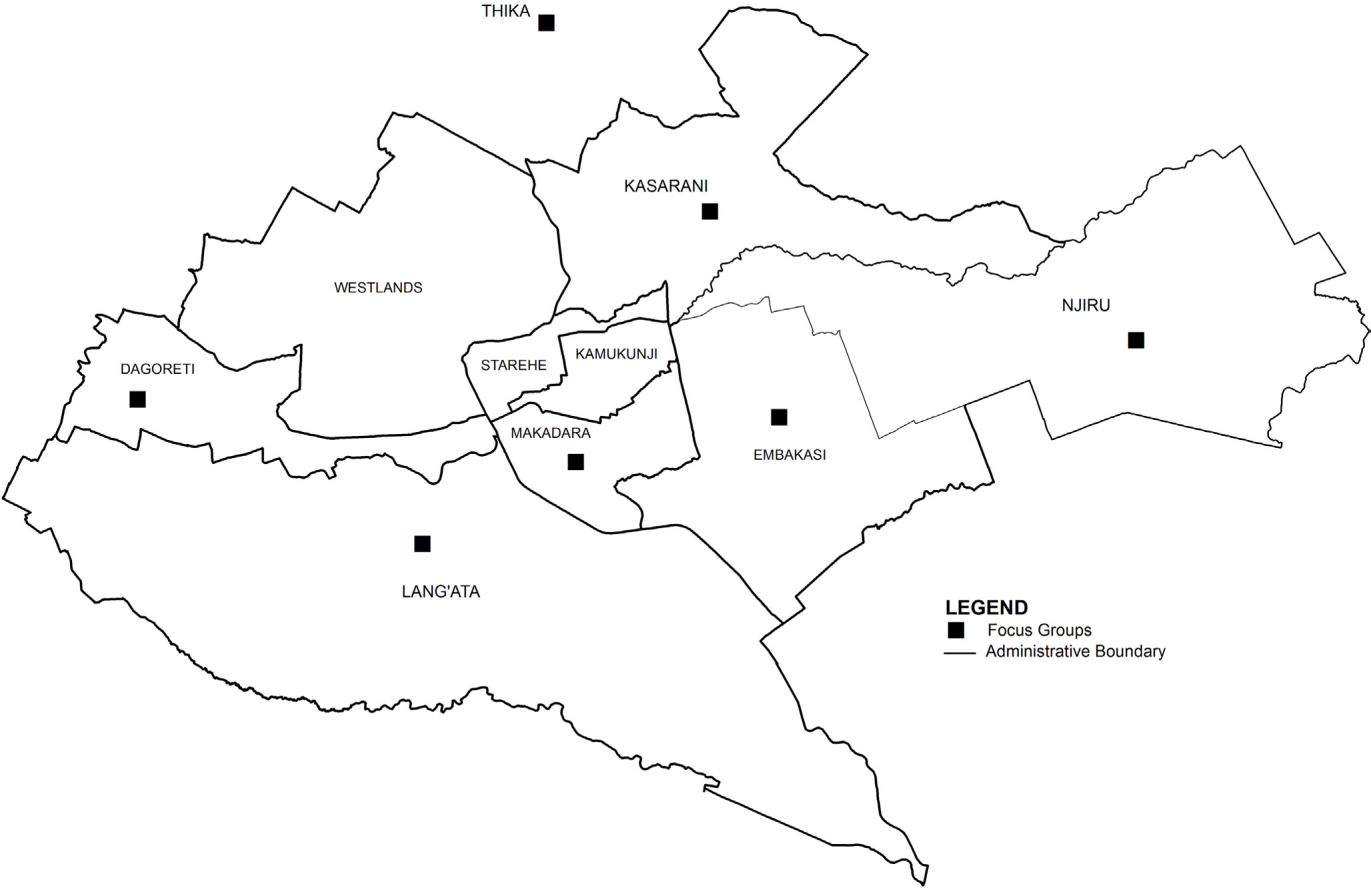
Division of former sub-counties in Nairobi, and indication of sub-counties where focus group discussions with livestock production officers were conducted.

A cross-sectional study was conducted in Nairobi County, Kenya, in 2013 and 2014. Six FGDs were conducted with LPOs from six Nairobi sub-counties (one separate FGDs per sub-county): Dagoretti (three participants), Lang’ata (four participants), Kasarani, Embakasi (both with five participants), and Njiru and Makadara (both with three participants). In addition, one FGD was conducted with a neighboring sub-county (Thika west—three participants) (Figure 1). These sub-counties represented the former district divisions. Kamukunji and Starehe sub-counties were not included in the study because they are located in the Nairobi’s business center and have minimal livestock production. Westlands district was not visited; Dagoretti was used as a proxy for this district. Overall, 23 participants (10 women and 13 men) LPOs participated in the FGDs. These belonged to a range of ethnic groups coexisting in Kenya (Kikuyu, Kamba, Luo, Kalenjin, Luhya, among others).

Research study ethical approval was obtained from the ethical committees from the International Livestock Research Institute (ILRI-IREC2014-04/1) and the Royal Veterinary College (URN 2013 0084H). Permission to do the study was granted by the Kenyan Ministry of Agriculture and Livestock Development. The FGDs were organized with the help of the chief LPOs of each sub-county, who facilitated assistance of the officers.

### Data Collection

The FGDs followed a semi-structured interview guide. The purpose of the study was explained prior to the FGDs to participants; these were requested to provide written consent. Subsequently, the officers were asked to enumerate and explain their roles and responsibilities as LPOs and the main challenges they faced as part of their everyday work. After this, the FGD activity was divided into seven parts; each of these focused on a specific livestock species (i.e., broilers, layers, indigenous chicken, pigs, beef cattle, dairy cattle, and small ruminants). Other livestock species were then also discussed if found relevant by participants in the area (e.g., dairy goats and rabbits).

For each livestock species, the following aspects were covered with the participants:

1. Identification of different production systems in their respective sub-counties: LPOs were given the freedom to categorize farms as they felt appropriate, but were also asked to categorize livestock keepers according to the size of production systems. Once the different systems were identified, LPOs were asked to estimate the proportion of livestock keepers falling under each type of production system. The classification was then drawn in a flipchart and was used as a basis for subsequent questions.
2. Mapping of existing supply chains: the main input sources (replacement stock, feed, and water) and output sources (animals and animal-derived products) used by the different systems were mapped. The proportion of livestock/product flowing through each supply chain for each livestock system was then estimated based on feedback provided by participants.
3. Identification and description of the relevant formal and informal animal health providers in each production system.
4. Description of the management practices of dead animals and the perception and experiences shared by participants concerning the most important food safety risks associated with each livestock production system.
5. Description of the main gender’s roles and responsibilities for each livestock production system.

The interview guide allow for flexibility and probing of the questions depending on the issues raised by participants (e.g., follow-up questions were possible if a food safety risk was mentioned by participants when discussing other sections, such as supply chain structure). The finalized interview guide used for the FGD can be accessed in the Supplementary Material Annex A. Quantitative data were obtained through achieving consensus on the adequate proportions and ranks given to each system, supply chain, or other factor. For this, participants were asked to agree or to provide a different estimate on the proportion obtained. When discrepancies emerged, the facilitator encouraged the discussion among participants to agree on final estimate. All seven FGDs were voice recorded and six of these were also video recorded after permission was given by participants. Each FGD lasted between 3 and 4.5 h.

### Data Analysis

Data collation and qualitative thematic analysis: all data were collated into templates using a customized Microsoft Word document. These templates presented the following coding structure, repeated accordingly for each livestock species (except the first two codes):

(1) LPO responsibilities,
(2) LPO challenges,
(3) Value chain functionality:
  - Type of livestock systems,
  - Source of replacement animals,
  - Source of feed and water,
  - Distribution of livestock and animal-derived products,
  - Challenges in the supply chains,
  - Livestock keepers associations or groups (formal or informal).
(4) Animal husbandry issues
(5) Gender issues,
(6) Use of different animal health providers,
(7) Management of dead animals,
(8) Food safety risks,
(9) Other factors.

Triangulation was followed for the purpose of this project, through an iterative process of careful analysis of the content of the memos produced by researchers during the FDGs with the respective audio/video recordings of these sessions. Relevant data were then collated and placed within each of the coding sections. Thematic analysis (9) of the data was then conducted to identify salient themes that provide an understanding of the factors associated with the codes described above. A theme may represent a perception reported by the participants about a given code (e.g., a perception on a given food safety risk) or could be a factor emerging from the discussions between the participants that the authors identified as relevant within a specific code (e.g., “Adult pigs are slaughtered in backyard areas without any inspection” reported during discussion of supply chain functionality and classified as a theme within food safety risks). This analysis was performed separately for each sub-county (or each FGD), in order to maintain association of salient themes with the relevant geographical area. Themes from all the areas were then compared to produce a narrative for each of the codes. In addition, salient themes related to animal health managers were plotted in diagrams to better visualize dissimilarities on the roles of these stakeholders across the different types of livestock production systems. The same team of researchers that conducted the FGDs also performed all transcriptions of relevant information from audiovisual records for consistency purposes. The thematic analysis was mainly performed by the first author. The emerging themes were reviewed by two co-authors who participated in the FGDs for validation purposes.

Supply chain mapping analysis: a mapping diagram that represented the overall urban farming supply chains in Nairobi was produced for each livestock species. For this purpose, the diagrams obtained in the focus groups (in the flipcharts) in combination with the salient themes related to chain mapping information collated through the transcription of the audiovisual recordings were combined. Mapping diagrams were drawn using SmartDraw version 4.1 (SmartDraw software Incorporated, San Diego, CA, USA). The use of FGDs to mapping food value chain systems was based on previous studies conducted by Alarcon et al. (10).

## Results

### Category of Livestock Farms in Nairobi

Classification of livestock keepers according to size of production is shown in Table 1.

**Table 1:** Number of livestock kept per livestock keeper category identified for each species in Nairobi (in brackets the proportion of livestock keepers with each species in each area).

Species	Farm type	Dagoretti	Njiru	Kasarani	Langa’ta	Makadara	Embakasi	Thika west
Dairy cow	Total animals	3,884	1,157	7,744	11,345	535	1,900	
	V. small	–	–	–	–	1 (40%)	–	–
	Small	1–2 (majority)	1–3 (30%)	2–5 (50%)	1–2 (70%)	2–3 (40%)	1–3 (65%)	1–5 (80%)
	Medium	3–20	4–10 (60%)	6–19 (25%)	3–5 (29%)	4–9 (20%)	4–10 (20%)	6–10 (18%)
	Large	–	11–50 (10%)	>20 (20%)	11–15 (1%)	10	11–20 (15%)	10–20 (1.5%)
	V. large	–	–	300 (5%)	–	–	–	>20 (0.5%)

Sheep and goats	Total animals	7,922	5,425	9,820	22,390	2,048	5,370	
	V. small		<5	–	–	–	–	–
	Small	2–3 (80%)	6–10 (20%)	2–3 (70%)	1–6 (80%)	5–10 (70%)	1–3 (30%)	1–3
	Medium	10–20 (20%)	11–20 (60%)	10–15 (10%)	7–12 (18%)	11–50 (30%)	4–10 (20%)	4–10
	Large		21–50 (10%)	20–30	–	–	11–100 (35%)	10–15
	V. large		50–500 (5%)	>100	200	–	100–500 (15%)	

Pigs	Total animals	4,911	3,334	16,136	?	1,269	3,660	
	Small	–	1–5 (35%)	2–10 (80%)	2–8	1–2	1–5 (25%)	1–10 (80%)
	Medium	–	6–50 (15%)	11–15 (30%)	9–15	3–5	6–20 (60%)	11–20 (15%)
	Large	5–49	>50 (15%)	50	16–40	6–10	21–50 (15%)	30–50 (5%)
	V. large	50–100	–	–	–	>20	100–500 sows	–

Rabbits	Total animals	3,087	3,361	9,352	6,380	5,666	2,350	
	Small	1–5 (majority)	1–5 (40%)	–	5–10 (28%)	1–5 (60%)	1–20 (60%)	–
	Medium	–	6–20 (50%)	–	11–30 (70%)	6–10 (20%)	21–100 (35%)	–
	Large	–	21–100 (10%)	–	50–100 (2%)	11–20 (10%)	101–200 (5%)	–
	V. large	–	–	–	–	20–60 (10%) – one farm 500	–	–

Broiler chicken	Total animals	252,273	16,435	39,950	274,062	17,600	22,000	
	Household	–	Ap. 20 (2%)	–	–	–	–	–
	V. small	–	–	–	–	10 (5%)	–	150–200 (10%)
	Small	–	100–250 (30%)	100 (20%)	100–200 (40%)	50–100 (15%)	50–100 (30%)	300–800 (80%)
	Medium	–	251–800 (60%)	200–250 (65%)	201–500 (40%)	100–200 (30%)	101–500 (70%)	500–3,000 (8%)
	Large	–	801–3,000 (8%)	6,000 (15%)	501–2,000 (20%)	200–500 (30%)	501–1,000	5,000–10,000 (2%)
	V. large	–	–	6,000–10,000	–	–	–	–

Layer birds	Total animals	13,016	13,789	59,605	23,006	15,000	30,500	
	Small	<300	200–500 (20%)	100–200	50–100 (50%)	<100 (30%)	20–100 (30%)	300–500 (80%)
	Medium	300–500 (majority)	501–1,000 (70%)	250–500	101–200 (30%)	100–200 (50%)	101–300 (70%)	500–1,000 (15%)
	Large	–	1,001–2,500 (10%)	100–600	201–500 (20%)	200–500 (20%)	–	>5,000 (5%)
	V. large	10,000 (1 farm)	–	–	–	–	–	–

Indigenous birds	Total animals	41,177	34,669	86,656	20,071	14,500	35,500	
	Household	5 (100%)	1–5 (20%)	1–10 (80%)	1–10 (70%)	2–10	3–10 (100%)	1–10
	V. small	–	–	–	–	–	–	–
	Small	–	6–20 (40%)	–	11–50 (25%)	10–20	–	20–100
	Medium	–	21–50 (30%)	20–50 (20%)	51–100 (5%)	–	–	–
	Large	–	51–200 (10%)		>100 (1 farm)	200 (1 farm)	–	–
	V. large	–	–	–	–	–	–	–

### Dairy Cattle Keeping

#### Categories of Dairy Cattle Keepers

According to the participants, small-scale farmers with 1–3 animals represented the majority (50–80%) in the city, and were the only type of dairy system reported in informal settlements. Slums were estimated to harbor about 5% of dairy animals in a sub-county. Medium and large farms in the city had between 4 and 20 dairy cattle. Small-scale farmers had an estimated average production of 9–10 l per cow per day, while medium- and large-scale farmers had an estimated average production of 20 and 25 l per day per cow, respectively.

#### Mapping of Nairobi Dairy Cattle Keepers Supply Chain

**Figure 2:**
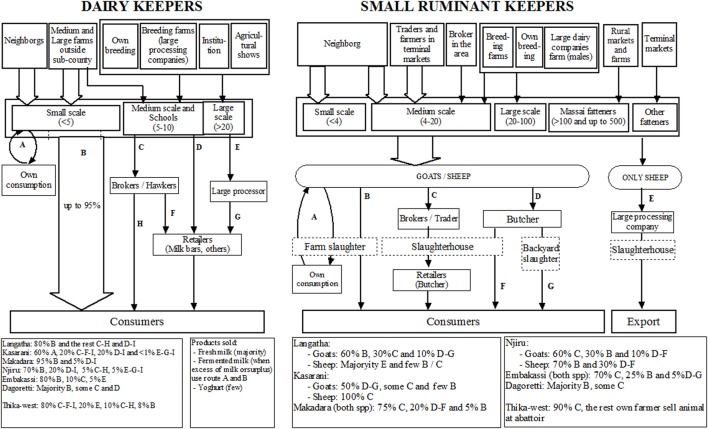
Supply chain mapping for the dairy urban keepers (left) and small ruminant urban keepers (right) in Nairobi. The box at the bottom of the figure shows the percentage of the overall flow of products (dairy) or animals (small ruminants) within each of the distribution chains identified in each sub-county.

The map of the supply chain used by Nairobi dairy farmers, as described by LPOs, is shown in Figure 2. Almost all milk produced was reported to be consumed locally and was believed to represent between 10 and 25% of milk consumption in the city (5% of consumption in informal settlements). Participants perceived that dairy farmers prefer selling their milk directly to consumers (60–95%) because it is “more profitable,” while consumers prefer buying from farmers because of “trust in quality,” “cheaper prices,” and its “easy access.” Furthermore, hawkers (street mobile vendors) were mentioned not to buy milk from local farms because of “high farm-gate prices compared to farms outside Nairobi.” However, in some informal settlements local farmers selling to hawkers were reported to be the prominent route of supply. The general pattern described was: small-scale farmers mostly selling directly to consumers; medium-scale farmers selling to processors, hawkers, hotels, and traders; and large-scale farms selling almost exclusively to processors. Several farmers in Nairobi also were reported to make fermented milk (“mala”) from the excess of milk, which was consumed in the family or to sell it to limited number of local consumers.

#### General Dairy Cattle Farm Management and Gender Characteristics and Challenges

A zero-grazing system was reported to be the most frequent, where farmers cut the grasses alongside the roads and collect vegetable leftovers, such as kales, cabbages, and maize stoves, from vegetable markets to feed the animals. Some farmers were described to purchase commercial feed from agrovet shops, while few farmers were able to make their own feed formulation. Medium-scale farmers in some areas were reported to buy hay and by-products from brewery companies and about 40% to have their own silage. Several dairy keepers were said to have contract with crop farmers to purchase Napier grass, or exchange it with manure. Large-scale farms were believed to have their own agricultural land outside Nairobi to feed their animals. It was reported that rarely dairy farmers kept a bull for reproduction purposes because of their high cost.

In small-scale farms, dairy animals were said to generally belong to the husband, who makes the major decisions such as buying or selling them. The women were described to be involved in managing the animals and selling the milk, and the money obtained to be shared with the husband. However, for medium- and large-scale farms, men were reported to dominate management activities with increased physical work, such as carrying feeds, while women were mainly involved in milking. In large-scale farms, all the activities were explained to be mostly done by men.

The LPOs perceived that one of the important challenges faced by dairy farmers was the “lack of land” to construct the recommended animal houses and to graze the animals. Many dairy farmers were described to have “poor and dirty structures” to keep their animals. In informal settlements, many dairy keepers were said to share the same house with their cattle, keeping these in the living rooms or in the bedrooms. “Insecurity” was another challenge identified by participants, with animals being stolen and sold to slaughterhouses. LPOs explained that farmers prefer building animal houses close to residential houses to protect animals from thieves. The “high price of feeds” was believed to be the main challenge faced by medium and large-scale farms.

### Beef Keeping

#### Category of Beef Keepers

It was estimated that most beef animals reared are Boranas or zebu. Beef farming in Nairobi was described as a “by the way production,” meaning that it normally happens as an aside to dairy or other activities. Six types of beef keepers were identified in Nairobi:

(1) *Maasai beef fatteners*: these were described as Maasai communities who keep large numbers of beef cattle (and also sheep and goats) in Manyattas (temporary traditional structures) mainly for fattening purposes (~3 month). They were reported to keep from 10 to 200 animals.
(2) *Other temporary beef fatteners*: these were described as traders who buy beef cattle from rural areas and fatten them for 1 month in areas near the slaughterhouses in Nairobi.
(3) *Animal transit keepers*: they were described as mainly pastoralists (mostly Maasai) who graze their animals along the roads and to come from neighbor counties outside Nairobi, such as Kajiado. They were said to come in search for pastures, especially during the dry seasons.
(4) *Keepers that come to slaughter*: these were identified by LPOs as people who bring their animals for slaughter or sale in the city terminal markets. However, in several cases, these animals were believed to end up staying for long periods in the area and some even to reproduce.
(5) *Beef farms*: in one sub-county, two beef farms with normal breeding activity were identified. Few institutions, such as schools, also were reported to rear beef cows.
(6) *Bull calves producers*: these were described as traders who buy small male calves at weaning from the dairy farmers and rear them. Also, some dairy farmers were said to keep few male calves and rear them for sale at one and half years old. It was estimated that these producers correspond to the majority of beef keepers in one sub-county (60%).

#### Supply Chain Analysis of Nairobi Beef Keepers

Participants reported that most animals from beef farms in Nairobi are sold to traders or brokers who slaughter the animals in the terminal markets that are close to their settlement. Some were said to be sold directly to butchers and retailers. In this case, animals were described to be also slaughtered in the terminal markets. In many settlements, such as the Maasai fatteners, the milk and mala (fermented milk) from beef cows was believed to be produced and consumed by them. Some beef producers in the city were mentioned to sell beef calves to finishing farms outside the sub-county.

#### General Beef Farm Management and Gender Characteristics

Most animals kept by Massai beef fatteners were reported to graze in informally organized pasture areas within the sub-county, along roads and river sides. In Embakasi, up to 1,000 beef cattle were estimated to be found in grazing areas near the abattoirs. LPOs believed that these keepers do not own the land, but that this normally belongs to the government. The bull calves were described to be mainly zero grazed (80%) or to be tethered outside to graze (20%). The zero-grazed animals were reported to be normally fed on “high-quality feed” from agrovet shops and/or with grass or hay cut along the road. Some beef keepers were said to have small gardens that they use to cut grass.

Participants perceived that men dominate all beef rearing and selling activities in the city, with the exception of beef calves born on dairy farms. In these farms, women were reported to mainly rear the animals, but the men to maintain the ownership and to sell them.

### Sheep and Goats Keeping

#### Category of Small Ruminant Keepers

The majority of small ruminant keepers were classified as small-scale (1–5 goats) and medium-scale farms (4–20 small ruminants), except in one sub-county where 50% of animals were reported to be clustered in large and very-large farms, with up to 500 sheep and goats per farm. Large farms with over 100 animals were described as Maasai temporary farms, but who could raise animals for up to 6 years. Transit keepers were also reported, corresponding to those that bring the animals to the terminal market or for grazing only. Fatteners, who are traders that buy animals in terminal market and fatten them for 2 month, were also identified. The ratio of goat to sheep varied depending on the size of the farms, with small farms keeping mostly goats and larger farms having an equal share of both species. In informal settlements, only small sheep and goat keepers were reported to exist. Most of these small-scale farmers were believed to keep small ruminant as a source for emergency funds.

The main breed of sheep in Nairobi farms were reported to be the Red Maasai and the Dorper. More purebreeds were said to be found in large farms, and a mixture of breeds in the medium- and small-scale farms. The main breeds of goats were the East African goat and the Galla goat.

#### Mapping of Nairobi Small Ruminant Keepers Supply Chain

Figure 2 also shows the supply chain associated with Nairobi small ruminant keepers, as perceived by LPOs. Several LPO focus groups had difficulties separating the food chains of goats and sheep. The reason reported was that many stakeholders sell sheep products, but label them as goat products. This was believed to be done because (1) “goats have higher demand than sheep in the market,” and therefore a higher value, (2) “consumers cannot differentiate between sheep and goat meat,” (3) “some consumers believe that goats carry fewer diseases,” and (4) “for some families sheep meat is a taboo.” Consequently, butchers were recommended to leave part of the goat tail in the carcass to facilitate identification. Sheep supply chains were reported to be directed to high income consumers or type of families with culture of eating these animals.

Backyard slaughter on the farm or homestead was reported as a frequent practice and associated with festive seasons and celebrations or ceremonies. For this, many consumers were believed by LPOs to prefer buying directly from Maasai keepers because their goats are perceived to have “better taste” as they are grazed in the forest. The main reasons provided for consumers to buy from local farms directly and do backyard slaughtering were (1) the “high prices in festive seasons” (it is cheaper to buy a live goat) and (2) the fact that this “helps them to have fun, learn how to slaughter and to teach their children.”

#### General Farm Management and Gender Characteristics and Challenges

Small farms were described to mainly feed their animals through scavenging, but also using vegetable markets waste and restaurant food leftovers. In two sub-counties, over 50% of small farms were estimated to raise their sheep and goats with zero-grazing practices. Most medium-scale keepers were reported to operate using a zero-grazing feeding regime. Large farms and fatteners were said to graze their animals in pastoral areas in the city. As with beef, Maasai keepers were described to raise and graze their animals in land owned by other people or the government.

Livestock production officers perceived men to dominate small ruminant rearing and selling among the Maasai keepers in the city. In other tribes and for small-scale farms, women were reported to be involved in rearing the animals because “these are easy to manage,” but men to be in charge of selling them. Participants believed that the main challenges faced by small ruminant keepers were the “lack of grazing area,” with grazing areas near the river being fenced; “insecurity and thieves,” especially in festive seasons with transit farmers more affected; and “animal diseases.”

### Pig Keeping

#### Categories of Pig Keepers

Small-scale farmers keeping 1–5 pigs (1–2 sows) were identified by LPOs as the most frequent system. These were reported to be mainly located in informal settlements (80% of pigs in Korogocho and almost all in Kibera). However, in Dandora dumping site, with 1,500 pigs, 65% of keepers were estimated to have between 6 and 50 pigs. Very-large farms composed of 100–500 sows were reported to exist in one sub-county. Around 70% of pig farmers in the city were described as farrow-to-finishing in the city, while the rest were finishing farms. Pigs were mentioned to be sold at a live weight of 70–90 kg and at 8 month of age for small and medium keepers, and at 5 month of age for large farms.

#### Mapping of Nairobi Pig Keepers Supply Chain

**Figure 3:**
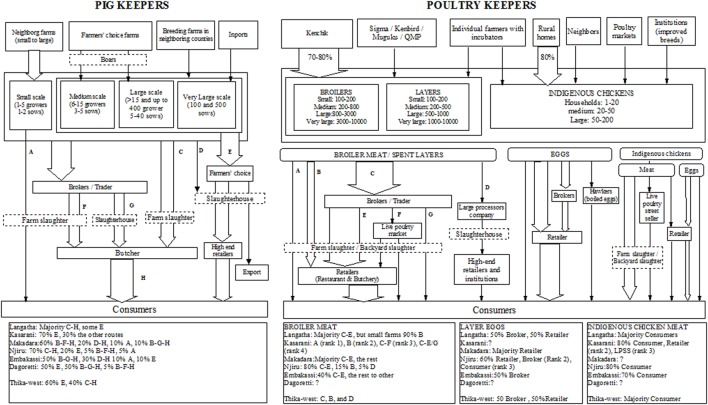
Supply chain mapping for the pig urban keepers (left) and poultry urban keepers (right) in Nairobi. The box at the bottom of the figure shows the percentage of the overall flow of products or animals within each of the distribution chains identified in each sub-county.

Figure 3 shows the supply chain associated with Nairobi pig keepers, as perceived by LPOs. Selling pigs to a large integrated company (Farmers’ choice) was reported as the most frequent chain in two sub-counties (50–70%) and to be minimal in other areas. It was mentioned that this company requires large number of animals per shipment and, therefore, is only accessible to medium to very-large farms. “Better prices” and “proximity to the company slaughterhouse” were also important factors perceived by participants for farmers to sell to this company. Selling live pigs to pork butcheries through brokers was reported to be mostly done by small- and medium-scale farmers in some sub-counties. However, in other sub-counties brokers were said not to be used because of the low prices they offered for pigs. In these areas, brokers were reported to be used only to sell dead pigs or pigs on the verge of death. Backyard slaughtering was described as a frequent practice due to “lack of abattoirs” existing in most sub-counties and the main route for pigs in informal settlements. In addition, some farmers were mentioned to sell pigs directly to consumers when they have gilts injured during mating.

#### General Farm Management and Gender Characteristics and Challenges

Feeding of pigs was dependent on the size of the farm and the area (peri-urban, urban, or informal settlement). Small-scale farmers were reported to largely depend on swill and market waste (e.g., sukumawiki, avocadoes, and fruits peeling) and to rarely include any commercial feed because of “lack of capital.” Free-range scavenging, with no commercial feed supplement, was described to be practiced by a large proportion of small-scale farmers (50–70%), especially in informal settlements. These farmers were mentioned to release their piglets at 7 weeks of age to scavenge with their mother. The medium-scale farmers were reported to use swill (from restaurants and schools) and commercial feed (mostly from agrovet shops).

Pig keeping in the city was perceived by LPOs a male dominated activity (mostly by youth), except in large farms where both genders were reported to be involved. The important physical activity required (e.g., pulling carts full of market waste) and the fact that pig keeping is done in a “dirty environment” were the main reasons identified by the participants for the lack of women operating in these systems. Women were said to be involved only on cleaning activities. Main challenges believed to be associated with pig keepers were: the “lack of pork abattoirs,” “scavenging pigs discourage consumption of pork,” “perception of being outlaws,” and “monopoly of pork in Kenya by [a big integrated company].” For the latter, it was reported that in times when this company stops buying pigs for one reason or another, many farmers ended up keeping their animals unsold for long periods.

### Poultry Keeping

#### Category of Poultry Keepers

##### Broilers Keepers

Medium-scale farmers keeping between 200 and 500 birds were perceived as the most common broiler system in Nairobi (60–70%). However, in informal settlements such as Kibera, about 80% were estimated to be small-scale farmers, with less than 100 birds. The “very large”-scale farms were reported to be owned by large processing companies. Farmers were also categorized as operating as “individuals” or in “commercial groups.” It was reported that many of these commercial groups form medium-scale farms by keeping birds together while maintaining individual ownership. These groups were believed to have been created “to obtain funding from the government,” “to get training services,” and “to improve access to markets.”

##### Layers Keepers

Majority of farms were estimated to keep between 100 and 300 layers. Medium and large farms were described to be run by individual farmers, women groups or institutions, such as secondary schools and churches.

##### Indigenous Chicken Keepers

Almost all indigenous chickens (65–100%) were reported to be owned by households, who have between 1 and 20 birds. Few large commercial farms with >50 to a maximum of 200 birds were mentioned to exist in several sub-counties.

#### Mapping of Nairobi Poultry Keepers Supply Chain

Figure 3 shows the supply chain associated with Nairobi poultry keepers, as perceived by LPOs.

Sourcing of poultry:

- *Broilers*: the large majority of broiler farmers in Nairobi (70–80%) were reported to source their day old chicks (DOCs) from one company. The “fast growth of birds” (5–6 weeks to mature compared to 6–7 weeks by other sources), “better feed conversion rates,” “lower mortality rates,” the company “good reputation,” and the “extra services” provided were identified as the main reasons for farmers to prefer this company. Other sources were believed to be used by some farmers because of their geographical proximity. In one sub-county, some farmers were mentioned to have incubators and to sell day old chickens to small-scale farms.
- *Layers*: one large company was identified as the major source of birds for the medium- and large-scale farms in Nairobi (70–80%). Small-scale farmers were reported to source birds from fellow farmers, medium-scale farms, and nearby agrovet shops. In one sub-county, small-scale farmers were reported to buy birds at point of lay (pullets) from the medium-scale farmers, “to reduce feeding cost.” In another sub-county, some farmers were said to have their own hatcheries that served both their farms and neighboring small- and medium-scale farms.
- *Indigenous chicken keepers*: most household (about 80%) were reported to source their indigenous chickens from rural areas. Many were explained to be obtained in form of gifts when visiting relatives. Neighbors’ farmers and local markets were perceived as other important sources for indigenous chicken in the city. Some commercial farmers were reported to own hatching equipment, while some large-scale farms purchase improved breeds from recognized breeding farms.

Distribution of poultry/products:

- *Broiler*: the majority of farmers (up to 80%), especially medium-scale farms, were estimated to sell their broilers to brokers because “they have ready market,” which farmers were said to lack. However, several small-scale farmers were reported to sell directly to retailers because of higher prices. Large-scale farmers were described to sell their birds to large processing companies, who then sell to large hotels and institutions. However, these companies were reported to only buy birds from contracted farms. The legs and heads were described to be mainly distributed through brokers or given to staff as payment for slaughter services.
- *Layers*: LPO explained that eggs from Nairobi farms are sold to retailers or to brokers, who then sell them to retailers. However, brokers were perceived as the least preferred option by farmers because “farmers know the market” and “eggs are easy to carry in trays.” Hawkers, those informal mobile street vendors, were identified as the people who buy the crack eggs and boil them before selling to consumers. It was believed that production in some sub-counties cover about 20% of eggs consumption. For the spent layers, it was reported that Nairobi consumers do not differentiate broilers from spent layer meat, and these are then sold in a similar manner.
- *Indigenous chickens*: own consumption or selling of birds directly to consumers were the main chains reported. The “low production of indigenous chickens” and the “high demand” for their products were explained to create an “easy market access” for farmers and “little need to use brokers.” Only few brokers were said to be involved with indigenous chicken or eggs. They were stated to purchase only from desperate farmers or in festive seasons when demand is higher. Some farmers were mentioned to sell their birds to people who resell these live birds in roadside sheds or at city market.

#### General Farm Management and Gender Characteristics and Challenges

##### Broiler and Layers Keepers

Five important companies supplying feed to broilers and layer farmers were believed to operate. LPOs reported that farmers purchase feed through stockist and agrovet shops. It was believed that the raising number of feed millers has led to “poor feed qualities in the market” which has affected the level of egg production and broiler growth at the farm level. Large companies were also reported to supply feeds to farmers buying their DOCs.

Small-scale farms were described to be mainly operated by women and young woman groups; however, birds were perceived to be owned by men. Medium-scale farmers were said to be run by both genders, while large and very-large broiler farms were operated mainly by men. However, in large-scale layer farms both genders were described to be involved. Participants perceived that the main challenges associated with these system were: “brokers buying broiler per head, while selling to consumers/retailer per kg.,” “lack of price harmonization,” “lack of knowledge on management practices,” “lack of capital to get training,” “aflatoxins in feeds,” “lack of hygiene at slaughtering, with use of dirty environment and water,” and “poor waste disposal by new farmers.”

##### Indigenous Chicken Keepers

Scavenging was reported in all sub-counties, with birds released in the morning to scavenge and to return back in the evening. LPO described that household indigenous chicken keepers were mainly women, the medium-scale farmers were both youth and women, while large-scale farms were run by men. Youths were reported to engage in poultry farming because of “lack of other jobs.” In one area, large-scale farming was reported to be practiced by women groups. Main challenges reported by participants for indigenous chicken keepers were “massive disease outbreaks (e.g., Newcastle) due to lack of vaccination” and “thieves during festive seasons.”

### Other Livestock Keeping

#### Rabbit Keeping

Rabbit farming was reported to be gaining popularity in the city and was done by (1) Individual farmers, (2) Groups, or (3) Institutions, such as schools, colleges, and prisons. New Zealand white and California white breeds were identified as the most commonly kept by medium and large-scale farms, while Flamys breed were kept by small-scale farms because of their slow growth. The large-scale farmers were affiliated to “Rabbit Kenya,” the only rabbit association in the country, and to be mostly owned by institutions.

Medium- and large-scale farmers were reported to buy bucks from organizations, such as the International Livestock Research Institute, Ngong breeding station-National veterinary farm, Limuru agricultural center, Rabbit republic, and Limuru agricultural center. However, farmers were said to use their own females for breeding and small-scale farmers to buy their replacement animals from fellow neighbor farmers.

Rabbit keepers were estimated to mostly sell their animals directly to consumers or for own consumption (about 50%). However, in one sub-county 90% of farmers were believed to sell rabbits to retailers, such as butcheries and restaurants, through a network of brokers. Institutions were described to mainly keep rabbits for own consumption. Large-scale farmers were reported to also supply big supermarket in the city.

The large- and medium-scale farms were mentioned to feed their rabbits on commercial feeds (pellets), while small-scale farmers feed them on green weeds, grasses harvested from the roadsides and/or gardens, market, and kitchen vegetables leftovers. Rabbit keeping was described as an activity mostly done by woman and children, but large farms were mostly owned by men.

#### Dairy Goats Keeping

The majority of dairy goat farmers were described as small-scale farmers keeping 1–6 goats, while medium-scale farmers have up to 20 goats. Some medium-scale keepers were identified as institutions, such as women prisons. No large dairy goat farm was reported in the city. In several sub-counties dairy goat keepers were reported to be organized in groups, where farmers help each other in issues of breeding, production and marketing of products.

Many farms were said to source their dairy goats from renowned breeding centers/farms outside Nairobi, several of which are owned by NGOs. A few were mentioned to buy from fellow farmers in the neighborhood. About 75% of the goat milk produced in some sub-counties was estimated to originate from small-scale farmers and to be sold directly to consumers, with the rest used for own consumption. Medium-scale farms were reported to sell their milk mainly to hospitals, and some to other institutions such as colleges and private consumers. The female goats (does) that are replaced were said to be either slaughtered for home consumption or sold to livestock traders, who take them to abattoirs. Most dairy goats were described as enclosed zero-grazed systems, with animals fed on commercial feeds and grass supplementation from roadside grasses and market waste. The dairy goat farming was seen as mostly managed by women, but who need to seek their husbands’ permission to sell the animals.

### Disease Management and Health Managers

**Figure 4:**
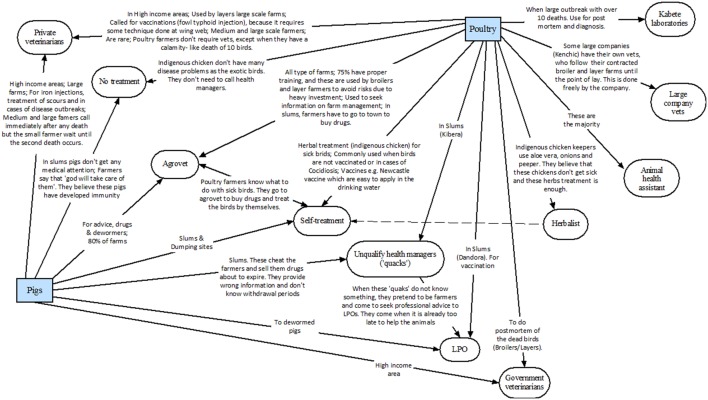
Use of different health managers by poultry and pig farmers in Nairobi, as reported by livestock production officers (LPOs).

**Figure 5:**
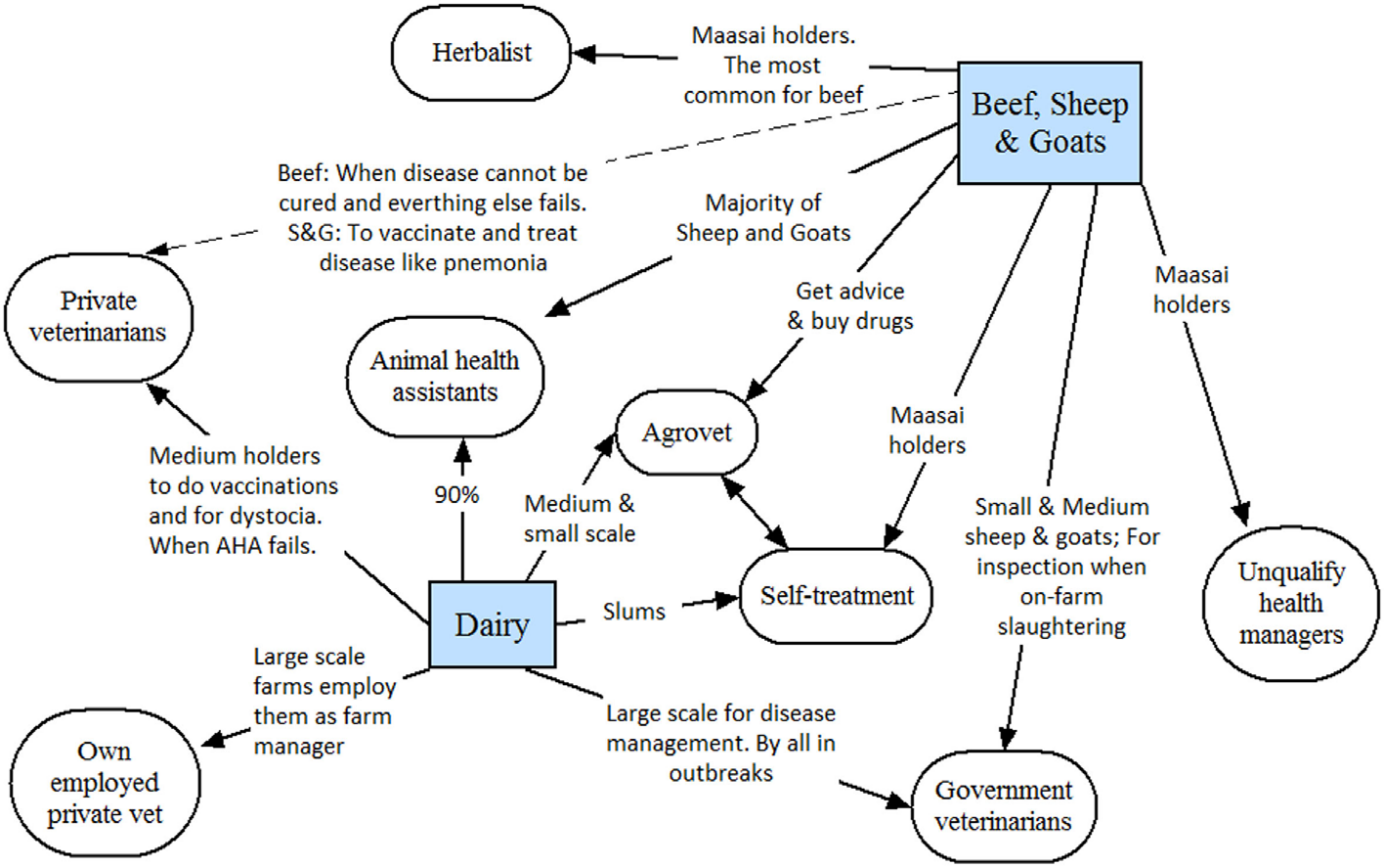
Use of different health managers by beef, small ruminant, and dairy farmers in Nairobi, as reported by livestock production officers.

Figures 4 and 5 shows the type of health managers used by each type of livestock keeper and the salient themes associated with each relationship. The health managers reported by LPOs were as follows:

- *Agrovets (Shops selling drugs and animal feeds)*: these were reported to be used by all type of livestock keepers for the supply of drugs, but also to obtain free advice on disease management. However, it was estimated that about 30% of staff working in these agrovet shops lack proper training and that in some cases “farmers are cheated” with wrong information/advice and by selling them drugs about to expire.
- *Unqualified health managers (“quacks”)*: these were reported to be mostly used by livestock keepers in informal settlements. LPO explained that these people, however, “claimed to be trained on animal health management.” When these “quacks” are faced to a disease situation that are not familiar with, they were said to “visit the LPOs for advice and by pretending to be farmers.” Quacks were considered “responsible for high prevalence of animal diseases.”
- *Herbalists*: were reported to be mainly used for beef and indigenous chicken keepers. For the latter, they were said to be used in non-vaccinated birds and to treat coccidiosis. Herbalists were considered to lack of any formal animal health training.
- *Animal Health Assistants or “veterinarian paraprofessionals”*: these were described as certificate holders with animal health training from an official institution. They were described also as private agents and to be used by the majority of commercial poultry producers and dairy keepers. It was estimated that they represent about 90% of trained people giving animal health care to farmers.
- *Livestock Production Officers*: these were reported to be used by poultry keepers in informal settlements and pig keepers for issues such as deworming and vaccination. The LPOs explained that they are also frequently used by rabbit keepers as the first call for disease issues.
- *Government veterinarians*: these were considered to be mainly used for inspection of animals slaughtered on farm, by dairy farmers in case of disease outbreaks, and by rabbit keepers when LPOs cannot handle the disease condition.
- *Private veterinarians*: they were reported to be rare and expensive, and mostly used by medium and large farms, and keepers in high income areas. They are considered to be used “when treatment fails on valuable animals” or “when there are large number of deaths.” Medium-scale dairy farmers were said to use them also “in cases of dystocia and for vaccination.” Some large companies were reported to employ them as farm managers. Dairy goat farmers were mentioned to use them frequently because of the high value of these animals.
- *Kabete laboratories (government diagnostic service)*: it was reported to be used by poultry farmers when there is “disease outbreaks with more than 10 deaths.”
- *No treatment*: many pig keepers in informal settlements and indigenous chicken keepers were considered to do not treat their sick animals, as they believe these are “resistant to disease” or fail to recognize disease in their animals.
- *Own-farmer treatment*: reported to be mostly done by dairy and pig farmers in the slums, Maasai beef keepers and indigenous chicken keepers. About 10% of livestock keepers were believed to provide self-treatment when animals get sick.

#### Management of Dead Animals and Main Food Safety Risks

Salient themes associated with management of dead animals and to existing food safety risks are shown in Table 2.

**Table 2:** Themes related to the management of dead animals and existing food safety risks in urban livestock farms in Nairobi, as obtained from the focus group discussion with livestock production officers in Nairobi.

Species	Themes associated with management of dead animals	Themes associated with existing food safety risks
Dairy cow	Rarely thrown away; Black market where vets lives in slaughterhouses are threatened if they do not cooperate; 90% of dead animals on farms are eaten, with 60% passing through abattoirs; Only those with suspicion of anthrax or FMD are not eaten; Given to feed dogs, or to pigs or crocodile farms; Buried; Thrown to the roadside during night hours; Dairy farmers are literate and do not eat dead animals	Use of plastic containers for milk transport; Milk containers are cleaned only with water and no disinfection; Water used for cleaning of low hygienic quality; Small farmers do not feel responsible for food safety; Sick animals that do not respond to treatment are sent to the slaughterhouse; Farmers do not observe antibiotic withdraw period; poor personal hygiene of people in charge of milking; use of dirty equipment on farms; People doing the milking of cows do not have hygiene certificate; Adulteration of milk is done with water and drugs; Small farmers disposed the manure on the roads; In slums some farmers keep dairy animals inside the household (in their bedrooms); meat is left to dry in the sun; Many dairy animals are kept in dirty shelters, with poor structures, unhygienic conditions and in high populated areas
Beef, sheep, and goats	In slums, dead animals are sold to meat butcheries at low prices, but butcheries sell to consumers at normal prices; No perception of wrongdoing when selling dead animals for consumption; Maasai people know which are the diseases (e.g., anthrax and snake bites) where dead animals should not be eaten; Farmers test for anthrax by throwing a small piece of meat (from their dead animals) to the fire and wait to see if it jumps; The black market for dead animals (operated through a network of brokers) is powerful and organized; Brokers cheat farmers by telling them they will feed their dogs with the dead animals collected (while in reality they sell the meat to consumers); Vets get life threaten to stamp meat (from dead animals on farms) and accept collusion; Few people consume dead sheep because these are perceived to have more pathogens compare to goats; 60% of beef cattle found dead on farms are consumed; 50% of small ruminant found dead on farms are consumed and the rest buried; Small-scale small ruminant keepers burn or bury their dead animals; Cook the meat and sell it to dog owners; Dead animals in field are left for dogs and birds to scavenge on them	The Maasai and small-scale farmers slaughter their sick animals, mix their carcass with some herbs and consume it; Very sick beef cattle may be sent to slaughter quickly without any treatment; Sick animals that do not respond to treatment may be sent to the slaughterhouse (to enter the food chain); Maasai bring animals from outside Nairobi to graze in the city for up to 3 month until they are slaughtered and, therefore, can transmit diseases to other animals in the area; Farmers do not observe the antibiotic withdrawal period before taking the animals to the slaughterhouse; Beef keepers use antibiotics carelessly; Beef keepers do not notify the authorities of the presence of notifiable diseases
Pigs	In slums, dead pigs are sold secretly for consumption; Dead pigs parts are boiled and used to feed other pigs; In dumping site, dead pigs are eaten by the homeless people; Farmers do not eat dead pigs, as they fear them (their meat); Thrown to dumping site for other pig and vulture birds to scavenge on them; Farmers with land bury the dead pigs	In slums, sick pigs may be slaughtered and its meat sold; Farmers do not want to incur on extra costs of treatment of sick pigs; Adult pigs are slaughtered in backyard areas without any inspection; Meat inspectors cannot inspect all pigs that are home slaughter (about 5% are not inspected); Pig feeds that are collected from markets may be contaminated; Farmers like feeding their pigs in the dumpsite and these can therefore transmit pathogens to people through contact or consumption
Poultry	Indigenous dead chickens are thrown into dumping sites for dogs to eat; Vets come to do postmortem of dead birds; When massive deaths occurs (more than 50 birds), these are sold to pig farmers; Single dead birds are cooked and fed to dogs; Small and medium-scale farmers sell dead birds to consumers; In large-scale farms, dead birds are buried; Some people throw them onto the roadside; Layer and broiler farmers do not consume dead birds because of fears of getting sick	Slaughtering is done on farm without any inspection, except for large companies; Inspection at slaughter only done when selling to big outlets (supermarkets and large processors); Hygiene of the farms and of the birds are not inspected; Source and quality of water for slaughtering and washing of carcass cannot be verified; Water contamination at transport level; Antibiotic withdrawal period is not followed by some farmers; Farmers do not wait for sick animals to die, they eat them
Rabbits	Rabbit meat is not very popular, and dead animals are not eaten; Dead rabbits are fed to dogs; Dead rabbits are not eaten even in slums and dumping sites	No inspection of rabbits is done at slaughter except when selling to reputable retailers; There are minimal food safety issues with rabbits because these are fed relatively safe feeds; Farmers do not observe antibiotic withdrawal periods after treatment, and do this knowingly; Rabbits are housed in poor structures and with poor hygiene; Rabbit feed mixes with the urine and suffer from diarrhea

## Discussion

The existence of livestock keeping in the city responds to a series of factors related mostly to rapid urban growth and individual food security and income generating needs, which outpaces the growth of services and employment, resulting in the majority of urban dwellers being in the low income bracket and having limited purchasing power (3, 11, 12). This fast urban growth also reduces significantly food availability and accessibility, which is aggravated by the increasing number of wealthy consumers in the city competing for food purchases. Urban livestock keeping is, therefore, a source of food security that can release pressure on poor households (that spend 60–80% of income in food) and provide essential micronutrients to avoid malnutrition (7, 13–15). In this study, LPOs estimated that up to 25% of milk or 20% of eggs consumed originate from these urban farmers. Urban livestock is also a source of employment and income, and frequently used to pay for children’s schools fees (16, 17). On the other hand, the high demand for animal source foods and increasing number of wealthy investors in the city generates livestock enterprises that employ low income people (18). All these factors explain the large diversity in profiles of livestock keepers in the city observed in this study. This diversity ranges from small scale with 1–2 animals mostly based on own consumption to large-scale commercial farms (with 10,000 broiler, over 2,000 layers, 300 dairy cows, 500 sheep, and goats) located in the peri-urban areas. These urban livestock systems also exhibit a wide variation of management practices as they exploit a number of ecological niches. These management systems ranges from well-structured commercial farms to small zero-grazed systems, transit farmers, temporary keepers, Massai fatteners, and small informal keepers that let pigs, ruminants, or poultry to scavenge freely.

The dynamic change in Nairobi, with increasing population and booming real estate ventures is potentially impacting livestock keeping in the city (6). Many livestock farmers in former rural areas have now become part of the city. Decrease in land size has also resulted in farmers being restricted on the type and size of livestock keeping. Consequently, farmers in the city are changing to intensive poultry and pig farming and to produce alternative species, such as rabbits (8). This is a pattern that is also being seen elsewhere in rapidly developing countries, which need to meet the food security needs of a growing population. The increasing demand for poultry meat and dairy products combined with the lack of cold chain and rapidly perishable products are also the likely reasons for the large number of urban and peri-urban poultry and dairy farmers. Dairy farming was also reported to be sustained in Nairobi due to lack of trust of consumers to milk from traders. For this reason, and due to higher profitability as Omore et al. (19) also identified, almost 95% of their milk is sold directly to consumers. The main reasons for the increase in pig farming has been related to increased urban pork consumption, proximity to established breeders from a large company and closeness to the feed manufactures (8). However, LPO perceived that the important population of pigs kept in informal scavenging systems creates consumer aversion to pork consumption (as these are perceived as dirty animals and consumer do not trust their meat), presenting therefore an important barrier for its commercialization. Nonetheless, scavenging pigs, and indigenous chickens, are relatively easy to sustain due to lack of cost on feed and housing (pigs fed and live in dumping site areas) and could represent an important source of income and/or food security to their owners living in these settlements (20, 21). On the other hand, formal pig systems are hindered by the lack of pig abattoirs, feelings of being outlaws and the dominance of one large company. Beef and small ruminant production were reported mostly as fattening and short-term activities, and mainly associated with terminal markets in the city. Indigenous chicken and small ruminants small-scale systems were supported based on rural origin of urban dwellers and mainly for consumption in festive seasons. All these reasons in combination with the analysis of the supply chains help to explain the role, existence, and evolution of different livestock keepers in Nairobi.

Nairobi was designed originally, in its master plan in 1964, as a green city, with large open spaces, to facilitate malaria control. This was reported as an important historical factor that explains the growth of urban agriculture and livestock keeping in the city (7). However, urban livestock keeping is an activity that is usually unplanned and uncontrolled by the state (7, 15). The role of urban livestock production in food security and livelihood presents, therefore, important tradeoffs with risks of pathogens transmission and environmental contamination, exacerbated with rapid informal urban growth (4, 18). Human contact with livestock in Nairobi is potentially important based on informal systems that keep animals scavenging outdoors, living inside households or in close proximity to these, but also based on continuous movement of animals for grazing (especially by ruminant from terminal markets) or in transit within the city. In addition, many of the farms kept in zero-grazed systems are fed with market waste, swirl from restaurants, and/or grass cut on road sides, and therefore increasing movement of pathogens throughout the city. Supply chain analysis indicates large numbers of animals being slaughtered in the households or retailer backyards, with little inspection and generating possible environment contamination to humans, wildlife, and other urban livestock. Moreover, water and sewage systems in the city are not designed for livestock production. Nairobi rivers that are polluted by industrial effluents and human waste are used and contaminated by livestock (7, 17, 22). Furthermore, results in this study indicate important waste management hazards, with cadavers disposed on roads and in many occasions sold and/or consumed, with existence of organized black markets. Manure disposal also was reported to be dumped along roadsides by some farmers. Results on the use of health managers illustrate these problems, with many small livestock keepers not treating their sick animals and slaughtering them, doing self-treatment, or getting advice from untrained health managers. This potentially contributes to generate several of the food safety risks occurring and shown in Table 2. There is, therefore, important scope to generate policies and city planning that can regulate these practices and minimize pathogens transmission.

As consequence of these risks, Nairobi by-laws (dating from 1961) declare that livestock production within city boundaries is an illegal activity, which can only be licensed under specific strict conditions (7). However, law enforcement has reported to be weak (17) and contradictory (3). In this study, LPOs reported that livestock keepers are continuously “being harassed by the city council,” while other government officers (such as LPOs and Government Veterinarians) provide advice on how to start a farm and also on husbandry and disease management practices. This system dysfunctionality and conflicting structures have been described as a common pattern in developing urban cities, as “holistic solutions are not part of public administrators mandate nor these have been trained to do so” (18, 23). Furthermore, urban livestock is often seen as a sign of “backwardness,” with authorities remaining hostile to these activities and few central government policies supporting it (18, 23). The situation for livestock keepers become even more difficult in informal settlements, where conflicts are created with food vendors and other business due to livestock eating their products or contaminating their environment (22). In Nairobi, control of these livestock keepers was reported in this study to present an important challenge, due to their “outlaw” status in the city, that makes them to avoid contact with government officers and generate “lack of trust”; their “general lack of training”; the “farmers lack of financial capabilities,” especially those small scale and/or in informal settlements; and their “inaccessibility” due to “insecurity” of those located in informal settlements or because they are “temporal” or “transit” farmers and not always present or available. “Presence of NGOs that give money to farmers” was also another challenge reported, as these livestock keepers expect payment in training activities organized by the government. “Lack of funding,” “government understaffing,” and “officers lack of transport” were other factors mentioned related to poor regulation and training of livestock keepers. Since 2013, with the new constitution in Kenya and the devolution laws, Nairobi County has maintained the existing laws regarding urban livestock keeping and, hence, continue to be an illegal activity. However, LPO reported that attitude of the city council is currently changing as they “see them now as business and food security entities.” Currently, new policies that will designate “areas for livestock farming” were reported to be under consideration, but it is unsure if these would be effective. However, in the authors’ opinion, even though urban livestock could cause food safety and environmental risks, these could be taken care of through better management and educational programs. The importance of urban livestock to food security and livelihoods means that an outright ban should not be considered. Instead, policies aiming to educate farmers on the importance on animal and environmental health management and that can facilitate enforcement and access of government officer could potentially help to minimize risk practices occurring in urban farms. In addition, continuing understanding the role and challenges of the different livestock keeper is paramount for the implementation of policies.

Livestock production officers reported several gender differences in each of the urban livestock systems. In small systems of dairy cattle, small ruminant (including meat and dairy goats), poultry, and rabbit, women were perceived to have an important role in managing the animals. However, only for dairy cattle, dairy goats, rabbits, and indigenous chicken, women were also responsible for the selling of the animals. This may have implication on food security, as woman have been reported to better use the benefits to meet household food security needs, but also to have higher rates on unemployment (24, 25). Men were perceived to participate in the management of beef and pig systems, but generally also to maintain ownership rights in most of other livestock species systems. They were also reported to be more involved in managing animals in large-scale farms. These findings are consistent with other gender studies conducted in Kenya and Africa (26). This understanding of gender differences is critical for designing policies and interventions aiming at reducing food safety and disease risks, but also at improving food security and other potential social issues associated with urban livestock keeping.

The information generated in this study summarizes LPOs experiences, knowledge, and perception of the livestock situation in the city. This represents the main limitation of the study, as other peoples’ perceptions are not accounted for. Interpretation of the results, such as the existing food safety risks identified or the themes reported by LPOs on attitudes, behaviors, and beliefs of other stakeholders, should be interpreted with care, as larger field studies are needed to validate their representativeness. Some estimates obtained are, therefore, approximations on the overall patterns of livestock structure, supply chains, and disease management existing. Based on the size of Nairobi city, its diversity of settlements and the important population of urban livestock, this qualitative approach was required to understand the overall system. The role of LPOs in providing extension services (e.g., advice on housing, animal husbandry, hygiene, etc.) to farms and their contact with different types of livestock keepers situated them in an ideal position as key informants for this study. The results provided here represent a baseline structure that is useful to design future research focused on specific urban production systems or livestock species and that involves other stakeholders in the supply chain. These results are also useful for researchers and policy makers to further investigate and address potential issues on animal disease management and food safety risk practices of urban livestock keepers. Another limitation was the lack of time as many different species had to be investigated in each focus group. In this regard, information regarding multiple livestock species system could not be explored. Indeed, a common system observed in Nairobi is the combination of broiler, dairy, and pig keepers in peri-urban areas (authors’ personal observations). Guendel (17) estimated that 50% of livestock keepers in Nairobi keep only one livestock species. The keeping of other exotic species, such as quails, ducks, and turkeys, in the city is also becoming popular and should be considered in future research studies.

The results obtained here provide a powerful background that can be used as a basis to design future studies aiming to investigate in more details the different urban livestock systems and their disease risks. The information obtained here is also crucial for policies aiming to control urban livestock and their possible impact on zoonotic disease transmission, environmental pollution, and food security.

## Author Contributions

The manuscript was written and the data analysis was done by PA and PM. The Data collection was done by PA, PM, MM, JA, and SK. Data entry was done by PM, SK, and MM. The discussion and interpretation of the results was done by PA, JR, and EF. The study design was done by PA, JR, and EF.

## Conflict of Interest Statement

The authors declare that the research was conducted in the absence of any commercial or financial relationships that could be construed as a potential conflict of interest.

## Footnotes

^1^Crude biomass estimations based on data from the Ministry of Livestock and Development report 2013 (8) and using corresponding average adult live weights and half of this weight for young people and livestock. The estimation also assumes that 75% of humans are adults; 75% of dairy cattle, dairy goats, and layers birds are adults; and 50% of other livestock are adults.
