# Supplementary material for: Urban Livestock Keeping in the City of Nairobi: Diversity of Production Systems, Supply Chains, and Their Disease Management and Risks
Source: Front Vet Sci. 2017 Oct 25;4:171. doi: 10.3389/fvets.2017.00171 (PMC5669286; doi:10.3389/fvets.2017.00171)

**Urban Disease Emergence (URBAN-ZOO) Project**

**Protocol for focus group discussion to livestock production officers**

**Economic threat component**

**Royal Veterinary College**

**Before starting:**

At arrival each participant will be given a nametag, in order to facilitate discussion. Then the researchers will present themselves, the project and the purpose of a focus group. After this, the researcher will read the consent form to the group and will then ask the participants to sign it if they agree with the conditions. The focus groups will continue with those participants that have agreed to the conditions of the focus group. Permission will also be asked for the use of a camera recorder. If permission is not obtained, then only annotations will be recorded.

One facilitator will lead the focus group discussion and two assistant will take notes of the conversation.

**Introduction of participants:**

Facilitator to ask each participant to introduce himself or herself by indicating their role and area they work with.

**Section 1 – Questions related to LPO responsibilities**

1. Could you please explain the locations within the sub-county that you or this office is responsible for?
2. Please explain what your responsibilities as livestock production officers are?
   1. Prompts:
      1. Explain your day to day activities?
      2. How do you interact with livestock holders?
      3. Do you have any responsibility on animal health issues? Please explain?
      4. If interactions with other people/stakeholders is mentioned, ask how they interact with them.
3. What are your overall challenges with livestock holders in your sub-county? (facilitator to make a list of challenges mentioned in the flipchart)
   1. Why or how is this a challenge?

**The next sections (2-5) will be repeated for each livestock species: Beef, Dairy cattle, Pigs, Small ruminants, poultry broilers, poultry layers and poultry indigenous chickens. At the end, LPO will be asked if other relevant livestock species are significant in their sub-county. If so, the following sections will be repeated to obtained knowledge on their systems.**

1. Which are the most important type of livestock production in your sub-county?

**NEXT SECTION FOCUS ON ONE LIVESTOCK SPECIES**

**Section 2 – Question related to identification of systems**

1. In your views, what are the different systems of [LIVESTOCK SPECIES] exiting in your sub-county?
   1. How would you classify them?
      1. Please describe how these systems operate?
      2. Please explain what is the main purpose of these systems?
      3. Is there is other way to classify these farmers?
   2. And in terms of number of animals kept? How would you classify them?
      1. If needed, facilitator to explain that they could classify farms in small, medium or large if relevant for their area. Prompts:
         1. What would be a small [LIVESTOCK SPECIES] keeper in your area? What would be the range of animals in this system?
         2. What would be a medium [LIVESTOCK SPECIES] keeper in your area? What would be the range of animals in this system?
         3. What would be a medium [LIVESTOCK SPECIES] keeper in your area? What would be the range of animals in this system?
         4. Facilitator to identify if there could be very large or very small [LIVESTOCK SPECIES] keeper in your area? What would be the range of animals in these systems?
      2. Does any of the “size categories” overlaps/matches with the previous categories mentioned?
      3. Facilitator to draw in the flipchart boxes with different systems identified.
   3. What, in your view, is the proportion of [LIVESTOCK SPECIES] farmers in the different systems in your sub-county?
      1. Facilitator to use the flipchart and the boxes with the different systems to help participants to estimate proportions.
      2. Facilitator to ask other participants to agree or to provide a different view on the estimate given. Facilitator to write all the proportion estimates in the flipchart. Once all the estimates for all the different system are provided, facilitator to ask again participants if they would like to redistribute the proportions.
   4. Are there any of the systems identified more prominent in a specific area of your sub-county? (or clustered in an area)
      1. Prompt: Is any of these systems more prominent in informal settlements in your area? If so, how would the overall proportions changes in this area?
   5. Other specific prompts:
      1. Is there any differences in production performance between these systems?
      2. For sheep and goats: facilitator to ask participants the proportion of sheep or goats in the different type of farms.
   6. Are there any associations or groups of [LIVESTOCK SPECIES] farmers in your sub-county?
      1. Please describe these groups.
      2. What is their main role? Why these associations exists?
      3. If no associations or groups, ask their view on why?

**Section 3 – Questions to identification of supply chains and husbandry**

Using the flipchart, facilitator will use the next questions to create a flow diagram showing the sources of livestock and destination of livestock or livestock products to/from the different systems identified in the previous section.

1. What is the source of animals for the different [LIVESTOCK SPECIES] keepers?
   1. Is there any other source? Prompt if other specific sources are used: Independent farmers, households, rural farmers, rural households, neighbors, NGOs, government.
   2. How farmers do obtained animals from these sources? (prompt about any other middleman or agent involved)
      1. Facilitator to draw boxes of the different type of sources mentioned.
   3. Of all these sources, which is the most important sources?
      1. Facilitator to ask participants to estimate proportion of supply of the different sources if possible?
   4. Is any of the source associated with any of the type [LIVESTOCK SPECIES] keepers we have identified?
      1. Facilitator to draw arrows to indicate flows from different type of sources to the livestock systems identified in previous section.
   5. Why do you think there is this association?
   6. Why some [LIVESTOCK SPECIES] farmers use the different sources?
   7. What are the main challenges of [LIVESTOCK SPECIES] farmers associated to these sources?
2. What are the type of feeding and source of feed and water used by the different [LIVESTOCK SPECIES] keepers?
   1. Facilitator to ask participants to estimate proportion of different feeding systems if possible.
3. We would like now to draw the different chains coming out of these different [LIVESTOCK SPECIES] keepers. Could you please indicate what the different chains for distribution of animals or products from these farms are?
   1. Facilitator to draw the different chains in the flipchart
   2. Prompt for chains:
      1. Direct selling to consumers
      2. Selling to retailers
      3. Selling to large companies
      4. Use of slaughterhouse or backyard slaughter
      5. Use of middleman
   3. Which are the most common or most used chain in the sub-county?
      1. Please, estimate what is the proportion of chain used for the distribution/selling of animals//products in your subcounty? If proportion are not possible to estimate, ask participants to rank the chains in order of importance.
         1. Facilitator to write the proportion/rank of chains in the flipchart.
      2. What is the proportion of [LIVESTOCK product] consumed in your sub-county that originates from [LIVESTOCK SPECIES] keepers in the sub-county?
   4. Are there any chain more associated to different [LIVESTOCK SPECIES] keepers?
      1. Why farmers use these chains?
   5. What are the main challenges farmers faced with these chains?

**Section 4 – Questions about food safety risks**

1. Please indicate what are the type of animal health services/agents used by [LIVESTOCK SPECIES] keepers when they have a sick animal?
   1. Prompt about use of:
      1. Veterinarians
      2. Agrovets
      3. Herbalist
      4. Government vets
      5. Self-treatment or don’t use any
   2. Facilitator to list the type of animal health providers in the flipchart.
   3. Please explain which type of [LIVESTOCK SPECIES] keepers use the different providers of animal health services/agents identified?
   4. Why (or why not) do they use the different animal health agents?
      1. In which situations do they use these services?
2. Please indicate in your views, what are the major food safety risk practices of this [LIVESTOCK SPECIES] keepers?
   1. Prompt: Why is this a challenge/risk? Why does this challenge/risk exist?
   2. Please indicate which type of [LIVESTOCK SPECIES] systems is associated to this type of risk?
3. Please explain how [LIVESTOCK SPECIES] keepers managed their dead animals?
   1. Prompt:
      1. Is this different for the different production systems? Why?

**Section 5 – Questions on general challenges and gender issues**

1. Please estimate which gender predominates in the different systems identified?
   1. Why do you think there is this gender difference between systems?
   2. Is there separate role of woman and men in these livestock system?
   3. How this affect the interaction with people in the supply chain?
   4. Is gender associated with the different food safety risks identified?
2. What are the main other challenges faced by these livestock keepers?
   1. Prompt: Why is this a challenge?

**Once all species have been done, the focus group discussion will finalize. Thank the participants for their valuable help.**

Examples of flipcharts created in the focus groups:

Flipchart created on pig systems and supply chains during focus group with LPO in Embakassi
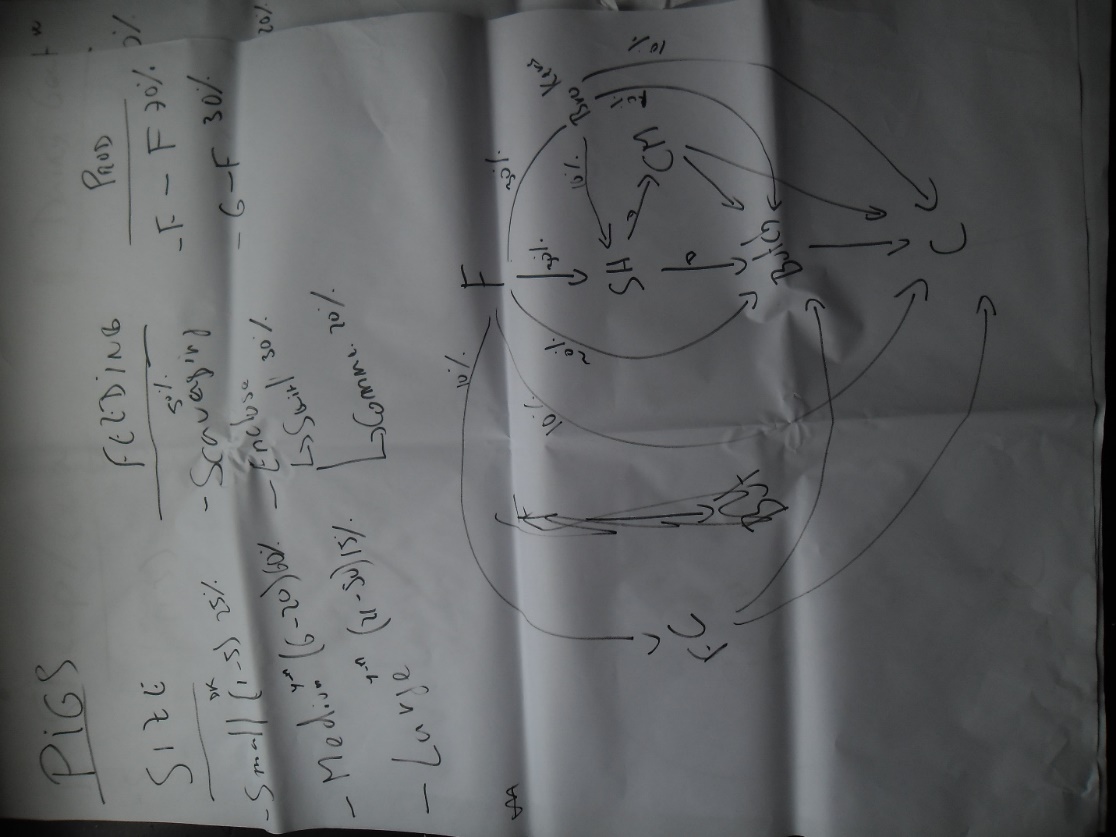


Flipchart created on pig systems and supply chains during focus group with LPO in Njiru
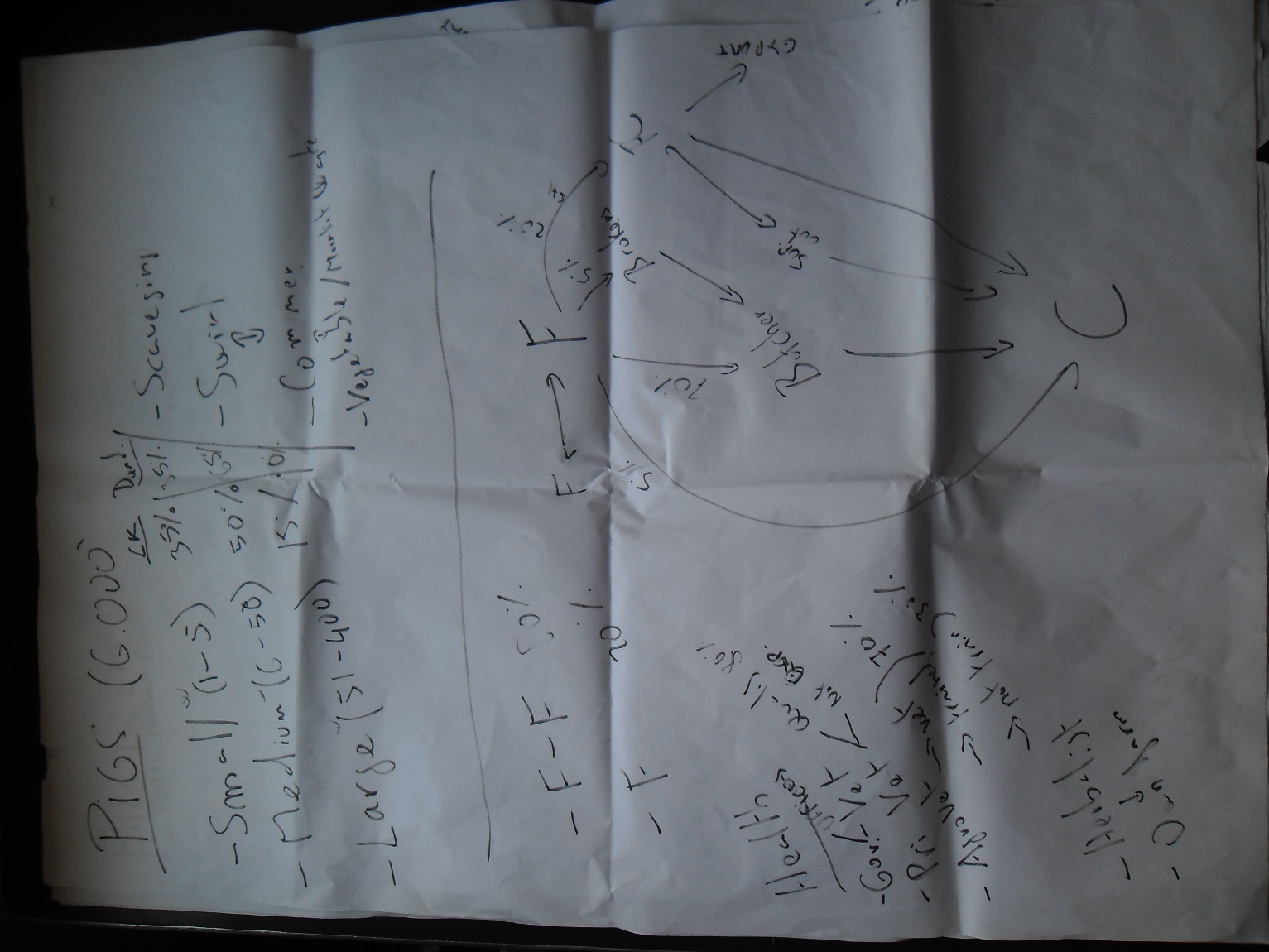

Supplement: Supplementary file 1 [file data_sheet_1.docx]
